# Coexistence of Two *bla*_NDM–__5_ Genes Carried on IncX3 and IncFII Plasmids in an *Escherichia coli* Isolate Revealed by Illumina and Nanopore Sequencing

**DOI:** 10.3389/fmicb.2020.00195

**Published:** 2020-02-13

**Authors:** Lang Yang, Yanfeng Lin, Lanfen Lu, Mei Xue, Hui Ma, Xuguang Guo, Kaiying Wang, Peihan Li, Xinying Du, Kezong Qi, Peng Li, Hongbin Song

**Affiliations:** ^1^Academy of Military Medical Sciences, Beijing, China; ^2^Center for Disease Control and Prevention, PLA, Beijing, China; ^3^Department of Laboratory Diagnosis, The Affiliated Zhongshan Hospital, Sun Yat-sen University, Zhongshan, China; ^4^Anhui Province Key Laboratory of Veterinary Pathobiology and Disease Control, Anhui Agricultural University, Hefei, China; ^5^The Sixth Medical Center of PLA General Hospital, Beijing, China; ^6^Department of Clinical Laboratory Medicine, The Third Affiliated Hospital, Guangzhou Medical University, Guangzhou, China

**Keywords:** coexistence of two *bla*_NDM–__5_ genes, *Escherichia coli*, transferability, Illumina short-read sequencing, MinION long-read sequencing

## Abstract

The emergence of carbapenem-resistant *Enterobacteriaceae* poses a significant threat to public health worldwide. Here, we reported a multidrug-resistant *Escherichia coli* strain with two different *bla*_NDM–__5_-carrying plasmids from China. Illumina short-read and MinION long-read whole genome sequencing were performed. Genomic analysis found that one *bla*_NDM–__5_ gene together with *mphA* was located on a 55-kb IncX3 plasmid, while the other *bla*_NDM–__5_ gene was on a novel 68-kb IncFII plasmid. Susceptibility testing and quantitative reverse transcription PCR results further indicated that the transconjugants with the IncX3 plasmid exhibited higher-level carbapenem resistance and expression of *bla*_NDM–__5_ than those with both plasmids or the IncFII plasmid. Two other β-lactamase genes (*bla*_*CTX–M–*__15_ and *bla*_OXA–__1_) were also detected on another 160-kb IncF plasmid. This is the first report of coexistence of two *bla*_NDM–__5_-carrying plasmids in a single bacterial isolate, highlighting the genetic complexity of NDM-5 carbapenemase circulation, and the urgent need for continued active surveillance.

## Introduction

Prevalence of carbapenemase-producing *Enterobacteriaceae* is a major concern around the world. NDM, one of the most important carbapenemases, represents a serious threat to patient care and public health. Since NDM-1 was first reported in 2009 (Yong et al., 2009), NDM-producing organisms has caused various types of clinical infections (Cornaglia et al., 2011). To date, 21 NDM variants have been discovered worldwide (Liu et al., 2018).

NDM-5, one of the most common variants encountered among *Enterobacteriaceae*, was first identified in an *Escherichia coli* isolate from a patient in the United Kingdom in 2011 with a recent history of hospitalization in India. It has two amino acid substitutions (Val88Leu and Met154Leu) in comparison with NDM-1, and confers increased resistance to extended-spectrum cephalosporins and carbapenems (Hornsey et al., 2011). Most strains usually harbored one single copy of the *bla*_ND__M_ gene. Coexistence of two copies of the *bla*_NDM_ gene is rarely detected. Recent studies reported the presence of two tandem copies of *bla*_NDM–__1_ in the chromosomes of an ST167 *E. coli* from China (Shen et al., 2017) and a *Pseudomonas aeruginosa* strain from Serbia (Jovcić et al., 2013). Duplication of the *bla*_NDM–__5_ gene on an IncF plasmid was observed in an *E. coli* strain (Feng et al., 2018), which showed identical carbapenem resistance levels to those conferred by an IncX3 plasmid with a single copy. However, no studies have reported coexistence of two *bla*_NDM–__5_ genes on different plasmids in the same strain. The effect of two *bla*_NDM–__5_-carrying plasmids on carbapenem resistance still remained unclear.

In the present study, we report an *E. coli* strain harboring two *bla*_NDM–__5_ genes, which were carried by IncX3 and IncFII plasmids, respectively. Susceptibility testing indicated that the transconjugant with the IncX3 plasmid alone showed the highest imipenem and meropenem resistance among all the transconjugants. Illumina short-read and MinION long-read whole genome sequencing was performed to gain an insight into the genomic and plasmid features.

## Materials and Methods

### Bacterial Isolation and Identification

The *bla*_NDM–__5_-positive *E. coli* strain GZ04-0086 was recovered from the stool sample of a patient through routine surveillance in Guangzhou, China, in 2018. The species level identification was performed using Vitek 2 compact system (bioMérieux, France). Presence of common genes encoding carbapenemases and extended-spectrum β-lactamases was determined by PCR screening (Balm et al., 2012; Zong and Zhang, 2013; Elumalai et al., 2014). The complete *bla*_NDM_ gene was amplified with previously described primers and confirmed by sequencing (Zou et al., 2015). Written informed consent was obtained from the patient. All experimental protocols were approved by the Institutional Ethics Committees of Academy of Military Medical Sciences.

### Southern Blotting, Conjugation Experiment, and Antimicrobial Susceptibility Testing

Bacterial genomic DNA from strain GZ04-0086 was prepared in agarose plugs and digested with the S1 endonuclease (Takara, Dalian, China). DNA fragments were separated by pulsed-field gel electrophoresis (PFGE) through a CHEF-DR III system (Bio-Rad, Hercules, CA, United States) for 15 h at 6 V/cm, with initial and final pulse time of 0.22 s and 26.29 s, respectively. The plasmid DNA was transferred to a positively charged nylon membrane (Solabio) and hybridized with the digoxigenin-labeled probe specific to *bla*_NDM–__5_.

Conjugation experiment was performed by filter mating using strain GZ04-0086 as donors and azide-resistant *E. coli* J53 as the recipient. The donor and recipient cultures were mixed at a ratio of 4:1 and collected on a membrane filter (pore size, 0.22 μm). The filter was placed on a BHI agar plate and incubated at 37°C overnight without shaking. The mixture was transferred into BHI agar plates containing 4 μg/ml meropenem and 200 μg/ml sodium azide. The transconjugants were selected after 72 h of incubation. The plasmid transfer frequency was calculated as the ratio of transconjugants to recipient cells. Presence of either one or both of the *bla*_NDM–__5_-carrying plasmids in the transconjugants was further confirmed by S1-PFGE.

Antimicrobial susceptibility testing of strain GZ04-0086 and the transconjugants was performed using Vitek 2 compact system following the manufacturer’s instructions. The results were interpreted according to the Clinical and Laboratory Standards Institute (CLSI) guidelines (CLSI, 2017). *E. coli* ATCC 25922 was used as the control strain. Minimum inhibitory concentration (MIC) values of imipenem and meropenem were further determined using MIC test strips (MTS, Liofilchem, Italy).

### Transcriptional Expression Analysis of *bla*_NDM–__5_

Transcriptional expression of *bla*_NDM–__5_ in strain GZ04-0086 and the transconjugants was determined by quantitative RT-PCR (qRT-PCR). Total RNA was isolated from bacterial cultures using RNApure Bacteria Kit (DNase I) (CWBio, China). RT-PCR was performed using UltraSYBR One Step RT-qPCR Kit (CWbio, China) in CFX96 Real-Time System (Bio-Rad, United States) with specific primers (NDM-RT-F 5′-GATTGCGACTTATGCCAATG-3′ and NDM-RT-R 5′- TCGATCCCAACGGTGATATT-3′) and 16S rRNA primers as previously described (Yu et al., 2019). Expression levels were normalized relative to the transcriptional level of the constitutive 16S rRNA and interpreted by the ΔΔ*C*_*t*_ method (Livak and Schmittgen, 2001). Statistical analysis was performed using a student t test to compare the log-transformed expression levels of *bla*_NDM–__5_ among strain GZ04-0086 and the transconjugants.

### Illumina/MinION Sequencing and Analysis

Genomic DNA was extracted from cultured bacterium using High Pure PCR Template Preparation Kit (Roche). Whole genome sequencing was carried out using both the Illumina MiSeq platform and the Oxford Nanopore MinION platform. The *de novo* hybrid assembly of short Illumina reads and long MinION reads was performed using Unicycler (v0.4.8) (Wick et al., 2017). Genome sequences were annotated using RAST (Aziz et al., 2008). The sequence type was determined through the MLST web server (Larsen et al., 2012). Genome sequences of seven *E. coli* ST44 isolates provided by BacWGSTdb (Ruan and Feng, 2016) were used to construct a Maximum-Likelihood phylogenetic tree using RAxML (v8.2.4) (Stamatakis, 2014). Plasmid replicon types and the sequence types of IncF plasmids were identified using PlasmidFinder and pMLST (Carattoli et al., 2014).

### Nucleotide Sequence Accession Number

The complete sequences of the chromosome of strain GZ04-0086 and plasmids pCTXM-GZ04, pNDM5-GZ04_B, pNDM5-GZ04_A, and p13k-GZ04 have been deposited in GenBank under the accession numbers CP042336 to CP042340, respectively.

## Results

### Microbiological and Genetic Features of Strain GZ04-0086

**FIGURE 1:**
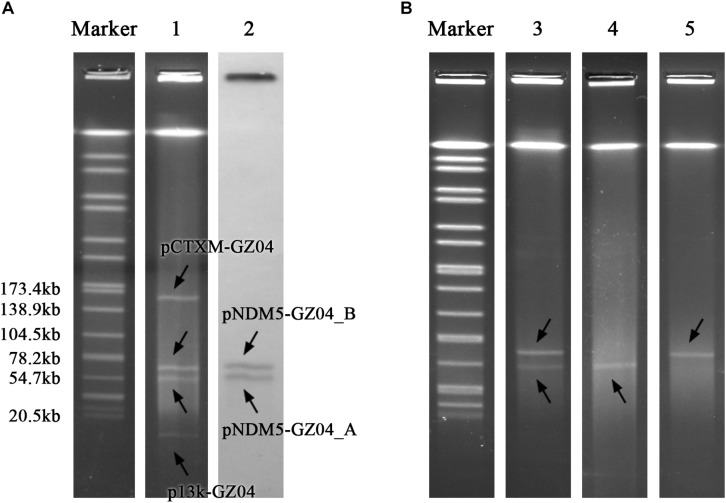
**(A)** S1-PFGE pattern for strain GZ04-0086 and southern blotting for the *bla*_NDM–__5_ gene. **(B)** S1-PFGE pattern for *E. coli* J53 transconjugants. Lanes: Marker, *Salmonella* serotype Braenderup strain H9812 as a reference size standard; 1, PFGE result for S1-digested plasmid DNA of strain GZ04-0086; 2, southern blot hybridization with the probe specific to *bla*_NDM–__5_; 3, 4, and 5, PFGE patterns for S1-digested plasmid DNA of *E. coli* transconjugants J53-AB, J53-A, and J53-B, respectively.

*Escherichia coli* strain GZ04-0086 was recovered from the stool sample of a patient in Guangzhou, China, in 2018. GZ04-0086 was resistant to most tested antibiotics including penicillins, cephalosporins, fluoroquinolones and carbapenems, but remained susceptible to amikacin, aztreonam, and trimethoprim-sulfamethoxazole (Table 1). PCR amplification and sequencing confirmed the presence of *bla*_NDM–__5_. S1-PFGE indicated that GZ04-0086 contained four different plasmids (<20 kb, ∼55 kb, ∼65 kb, and ∼160 kb) (Figure 1A). Interestingly, southern blotting further revealed the simultaneous presence of the *bla*_NDM–__5_ gene on the ∼55-kb and ∼65-kb plasmids (designated as pNDM5-GZ04_A and pNDM5-GZ04_B, respectively), which were both transferable to *E. coli* J53. The transfer frequency of the *bla*_NDM–__5_ gene was 1.07 × 10^–3^ per donor cell. Transconjugants containing pNDM5-GZ04_A (J53-A), pNDM5-GZ04_B (J53-B) or both plasmids (J53-AB) were obtained and confirmed by S1-PFGE (Figure 1B).

**TABLE 1:** Antibiotic susceptibilities of *E. coli* strain GZ04-0086, the *E. coli* J53 transconjugants and the *E. coli* J53 recipient.

Method	Antimicrobial	MIC (μg/ml)				
		
		GZ04-0086	J53-A^a^	J53-B^b^	J53-AB^c^	J53^d^
Vitek 2 system	Amikacin	≤2	≤2	≤2	≤2	≤2
	Ampicillin	≥32	≥32	≥32	≥32	≤2
	Ampicillin/sulbactam	≥32	≥32	≥32	≥32	≤2
	Aztreonam	≤1	≤1	≤1	≤1	≤1
	Nitrofurantoin	64	≤16	≤16	≤16	≤16
	Trimethoprim-sulfamethoxazole	≤20	≤20	≤20	≤20	≤20
	Ciprofloxacin	≥4	≤0.25	≤0.25	≤0.25	≤0.25
	Piperacillin	≥128	≥128	≥128	≥128	≤4
	Piperacillin/tazobactam	≥128	≥128	64	≥128	≤4
	Gentamicin	≥16	≤1	≤1	≤1	≤1
	Cefepime	≥64	≥64	16	≥64	≤1
	Ceftriaxone	≥64	≥64	≥64	≥64	≤1
	Ceftazidime	≥64	≥64	≥64	≥64	≤1
	Cefotetan	≥64	≥64	32	≥64	≤4
	Cefazolin	≥64	≥64	≥64	≥64	≤4
	Cefuroxime	≥64	≥64	≥64	≥64	≤1
	Tobramycin	≥16	≤1	≤1	≤1	≤1
	Imipenem	≥16	≥16	≥16	≥16	≤1
	Meropenem	8	≥16	8	≥16	≤0.25
	Levofloxacin	≥8	≤0.25	≤0.25	≤0.25	≤0.25
MIC test strip	Imipenem	16	≥32	12	16	0.125
	Meropenem	12	48	6	16	0.032

*^*a*^The *E. coli* J53 transconjugant harboring plasmid pNDM5-GZ04_A. ^*b*^The *E. coli* J53 transconjugant harboring plasmid pNDM5-GZ04_B. ^*c*^The *E. coli* J53 transconjugant harboring plasmids pNDM5-GZ04_A and pNDM5-GZ04_B. ^*d*^The *E. coli* J53 recipient.*

### Susceptibility Testing and *bla*_NDM–__5_ Expression Analysis of the Transconjugants

**FIGURE 2:**
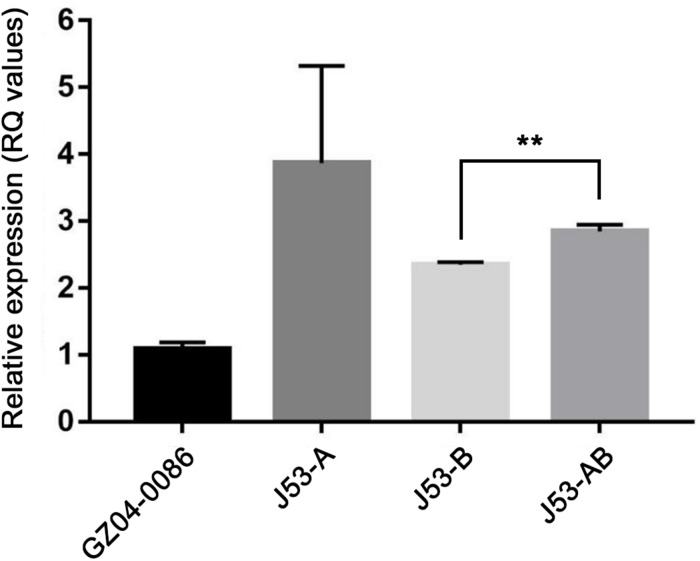
Relative expression of the *bla*_NDM–__5_ gene among strain GZ04-0086 and transconjugants J53-A, J53-B and J53-AB. Strain GZ04-0086 was used as the reference. Asterisks above lines indicate a statistically significant difference (** *P* < 0.01) as determined by Student *t* test.

All the transconjugants acquired resistance to ampicillin, ampicillin/sulbactam, piperacillin, imipenem, meropenem, and cephalosporins. However, decreased MICs of piperacillin/tazobactam, cefepime, cefotetan and meropenem were observed in transconjugant J53-B compared with those of J53-A and J53-AB. Interestingly, susceptibility testing using MIC test strips revealed that J53-A had the highest MICs of imipenem (≥32 μg/ml) and meropenem (48 μg/ml) among all the transconjugants. J53-AB had MICs of 16 μg/ml for imipenem and meropenem, while J53-B had lower MICs (imipenem, 12 μg/ml; meropenem, 6 μg/ml). The results of qRT-PCR also indicated that the expression of *bla*_NDM–__5_ in J53-A was 1.37-fold higher than that in J53-AB and 1.56-fold higher than that in J53-B (Figure 2). These slightly varied expression levels of *bla*_NDM–__5_ were consistent with the imipenem and meropenem MICs in all the transconjugants. Raw sequence reads mapped to regions specific to plasmids pNDM5-GZ04_A and pNDM5-GZ04_B revealed an average read depth of 251× and 198×, respectively, which implied corresponding copy numbers of *bla*_NDM–__5_-carrying plasmids in *E. coli* GZ04-0086 (Supplementary Figure S1). However, strain GZ04-0086 exhibited a relatively low transcriptional level of *bla*_NDM–__5_ compared with all the transconjugants but still had an imipenem MIC of 16 μg/ml and a meropenem MIC of 12 μg/ml.

### Genomic Insights Through Short-Read and Long-Read Sequencing

Strain GZ04-0086 was subjected to whole genome sequencing using both the MiSeq and the MinION platforms. Complete sequences of a 4.81-Mb chromosome and four plasmids were obtained through a *de novo* hybrid assembly. GZ04-0086 belonged to phylogenetic group A and sequence type ST44. *E. coli* ST44 has been frequently isolated from clinical settings (Kanamori et al., 2017; Runcharoen et al., 2017; Sonda et al., 2018). A phylogenetic analysis of *E. coli* ST44 revealed that GZ04-0086 was closest to isolates DO40 from canine and RDK06_431D from a patient in Tanzania, with 51 and 54 SNPs, respectively (Supplementary Figure S2). The *bla*_*CTX–M–*__15_, *bla*_OXA–__1_, *tet*(*B*), *tet*(*D*), and *aac*(*6*′)-*Ib*-*cr* genes were found on a 159,610-bp IncF [F36:F31:A4:B1] plasmid designated as pCTXM-GZ04. Another plasmid (designated as p13k-GZ04) had a length of 12,602 bp and was typed as Col440I (97% coverage and 93% identity).

### Genetic Characterization of *bla*_NDM–__5_-Carrying Plasmid pNDM5-GZ04_A

**FIGURE 3:**
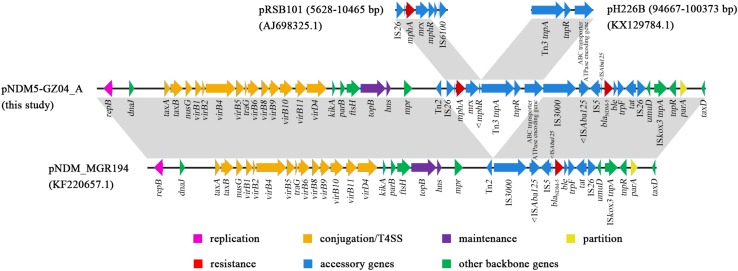
The comparative schematic diagram of plasmids pNDM5-GZ04_A, pNDM_MGR194, and fragments of pRSB101 and pH226B. The open reading frames are denoted by arrows. Regions with >99% identity among different plasmids are indicated by light gray.

The *bla*_NDM–__5_-carrying plasmid pNDM5-GZ04_A had 55,253 bp in length. The plasmid belonged to the incompatibility type IncX3. A BLAST search against NCBI revealed that pNDM5-GZ04_A presented 84% query coverage and 99% identity with plasmid pNDM_MGR194 in *K. pneumoniae* from India (Krishnaraju et al., 2015; Figure 3). Both plasmids shared the same backbone sequence containing genes for plasmid replication (*repB*), conjugation/T4SS (*virB* genes), maintenance (*topB* and *hns*) and partition (*parA*), and the genetic structure of the *bla*_NDM–__5_ gene, which was organized as IS*3000*-IS*Aba125*-IS*5*-*bla*_NDM–__5_-*ble*-*trpF*-*tat*-Δ*ctuA1*-IS*26*. The accessory region between *mpr* and *umuD* genes of pNDM_MGR194 was composed of a Tn*2* and the *bla*_NDM–__5_ genetic structure, while that of pNDM-GZ04_A consisted of a Tn*2* and an 18-kb novel composite transposon Tn*6638*. Tn*6638* was flanked by two IS*26* elements in the same orientation. In addition to the *bla*_NDM–__5_ genetic structure, a 5.7-kb fragment of a Tn*3* family transposon and a 3.2-kb remnant of an IS*26*-*mphA*-*mrx*-*mphR*-IS*6100* region were also carried by Tn*6638*. The 5.7-kb fragment contained Tn*3 tnpA*, *tnpR* and an ABC-transporter-ATPase-encoding gene, which was previously present in a Tn*3* family transposon in plasmid pH226B from *E. coli* (Zurfluh et al., 2016). The 3.2-kb remnant had undergone the deletion of the IS*6100* and the truncation of the *mphR* gene compared with the prototype *mphA*-carrying region in plasmid pRSB101 (Szczepanowski et al., 2004).

### Genetic Characterization of *bla*_NDM–__5_-Carrying Plasmid pNDM5-GZ04_B

**FIGURE 4:**
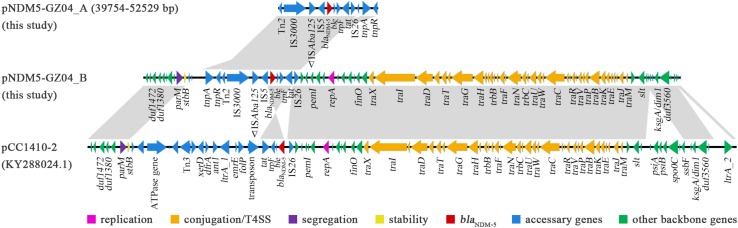
The comparative structures of plasmids pNDM5-GZ04_B, pCC1410-2, and fragment of pNDM5-GZ04_A. The open reading frames are shown by arrows. Regions with > 99% identity among different plasmids are denoted by light gray.

The other *bla*_NDM–__5_-carrying plasmid pNDM5-GZ04_B had a size of 68,545 bp and belonged to the incompatibility type IncFII [F2:A-:B-]. A BLAST search revealed that pNDM5-GZ04_B showed 84% query coverage and 99% identity to another *bla*_NDM–__5_-carrying plasmid pCC1410-2 in *K. pneumoniae* from Korea (Shin et al., 2017) but differed significantly from pNDM5-GZ04_A (Figure 4). However, pNDM5-GZ04_B shared the same *bla*_NDM–__5_ genetic context (IS*3000*-IS*Aba125*-IS*5*-*bla*_NDM–__5_-*ble*-*trpF*-*tat*-Δ*ctuA1*-IS*26*) with pNDM5-GZ04_A, while pCC1410-2 had a different context organized as IS*26*-*bla*_NDM–__5_-*ble*-*trpF*-*tat*. The 52-kb backbone of pNDM5-GZ04_B contained a set of core genes for plasmid replication (*repA*), conjugation/T4SS (*tra* and *trb* genes), stability (*stdB*) and segregation (*parM*). This backbone was highly identical to those of F2:A-:B- plasmids (Pu et al., 2016; Nahid et al., 2017; Shin et al., 2017) including pCC1410-2 but lacked a 5-kb region carrying the *psiA*, *psiB*, *spo0J* and *ssbF* genes associated with plasmid SOS inhibition, partition and recombination.

## Discussion

Emergence of NDM-producing *Enterobacteriaceae* represents an additional challenge for clinical management and infection control. A variety of plasmids have contributed to the dissemination of the *bla*_NDM_ gene among diverse *Enterobacteriaceae* species throughout the world. pNDM_MGR194-like IncX3 plasmids have been found to be a major vehicle for *bla*_NDM–__5_ (Wu et al., 2019). It was likely that pNDM5-GZ04_A had evolved from these pNDM_MGR194-like plasmids and undergone horizontal gene transfer which resulted in the additional insertion. Plasmids pNDM5-GZ04_A and pNDM5-GZ04_B had different backbones but the same *bla*_NDM–__5_ genetic structure, suggesting possible occurrence of similar transposition events. It was noteworthy that a second *bla*_NDM–__5_ gene could not be detected based on the Illumina short reads alone due to the repeated appearance of the same *bla*_NDM–__5_ genetic context. MinION sequencing played a crucial role in characterizing the coexistence of two *bla*_NDM–__5_ genes.

Despite the presence of *bla*_NDM–__5_, the transconjugant J53-B exhibited significantly lower carbapenem resistance than that of J53-A. This decrease might be associated with the lower copy number of pNDM5-GZ04_B. Our analysis also revealed a lower copy number of pNDM5-GZ04_B, which might be the key reason for the reduced expression of *bla*_NDM–__5_ in J53-B. Previous studies demonstrated that two copies of *bla*_NDM–__5_ in the same plasmid would not lead to stronger carbapenem resistances (Feng et al., 2018), while coexistence of two plasmids harboring *bla*_NDM–__1_ and *bla*_*KPC–*__2_, respectively, conferred higher resistances (Wu et al., 2016, 1). However, GZ04-0086 and J53-AB also showed lower carbapenem resistance than that of J53-A. The coexistence of these two *bla*_NDM-5_-carrying plasmids showed no additive effect on imipenem and meropenem resistance levels. The varied copy numbers and inter-plasmids competition seem to offset each other on carbapenem MICs and expression level of *bla*_NDM–__5_, which still need further investigation.

## Conclusion

In summary, our study characterized a multidrug-resistant ST44 *E. coli* harboring two *bla*_NDM–__5_ genes, which were carried by different plasmids. However, coexistence of the two *bla*_NDM–__5_-carrying plasmids did not lead to stronger carbapenem resistance. Both plasmids were transferable and might serve as new reservoirs for further dissemination of *bla*_NDM–__5_. Our findings highlighted the threat of NDM-5 carbapenemase circulation and the urgency of stringent surveillance and control measures.

## Data Availability Statement

The datasets generated for this study can be found in the NCBI GenBank, CP042336, CP042337, CP042338, CP042339, and CP042340.

## Ethics Statement

The studies involving human participants were reviewed and approved by the Institutional Ethics Committees of Academy of Military Medical Sciences. The patients/participants provided their written informed consent to participate in this study.

## Author Contributions

LY, YL, and LL performed the experiments. LY, MX, and HM analyzed the data. XG collected the samples. KW and PHL performed library construction and genome sequencing. HM and XD prepared the tables and figures. LY and PL prepared the manuscript. KQ, PL, and HS designed the study and revised the manuscript. All authors read and approved the final manuscript.

## Conflict of Interest

The authors declare that the research was conducted in the absence of any commercial or financial relationships that could be construed as a potential conflict of interest.
