# Supplementary material for: Coexistence of Two blaNDM–5 Genes Carried on IncX3 and IncFII Plasmids in an Escherichia coli Isolate Revealed by Illumina and Nanopore Sequencing
Source: Front Microbiol. 2020 Feb 13;11:195. doi: 10.3389/fmicb.2020.00195 (PMC7031209; doi:10.3389/fmicb.2020.00195)
Supplement: Supplementary file 1 [file Data_Sheet_1.docx]

Supplementary Material

## Supplementary Figures

**
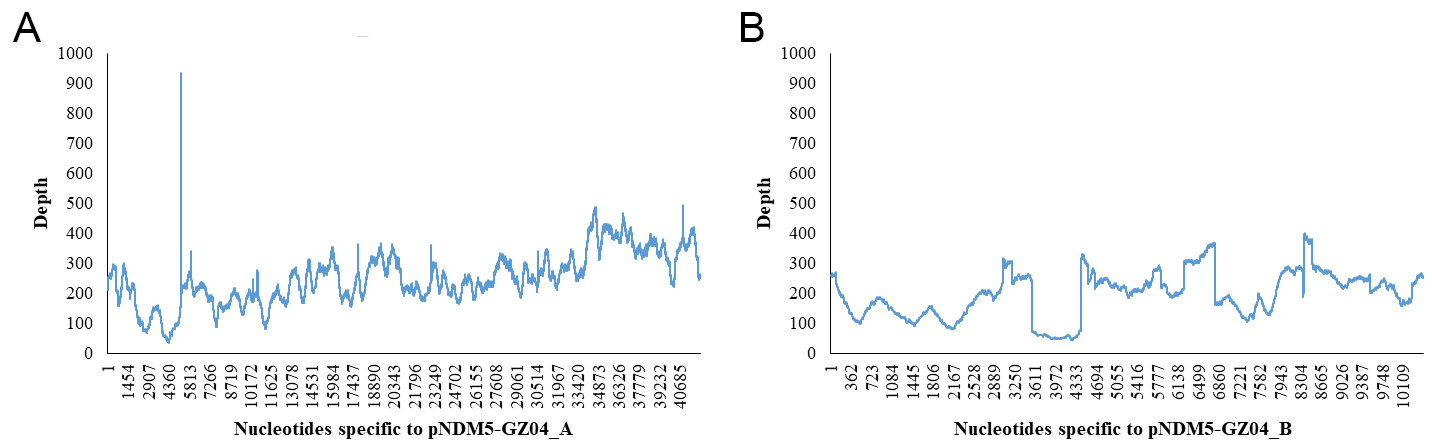
**

**Figure S1. The distribution of read depth among regions specific to (A) pNDM5-GZ04_A and (B) pNDM5-GZ04_B.** Read depth was computed per nucleotide by mapping the raw sequence reads to plasmid sequences. The regions specific to each plasmid were identified by aligning the plasmid sequence against the sequences of the chromosome and other plasmids of GZ04-0086. The distribution of read depth was shown with the specific regions being concatenated.

**
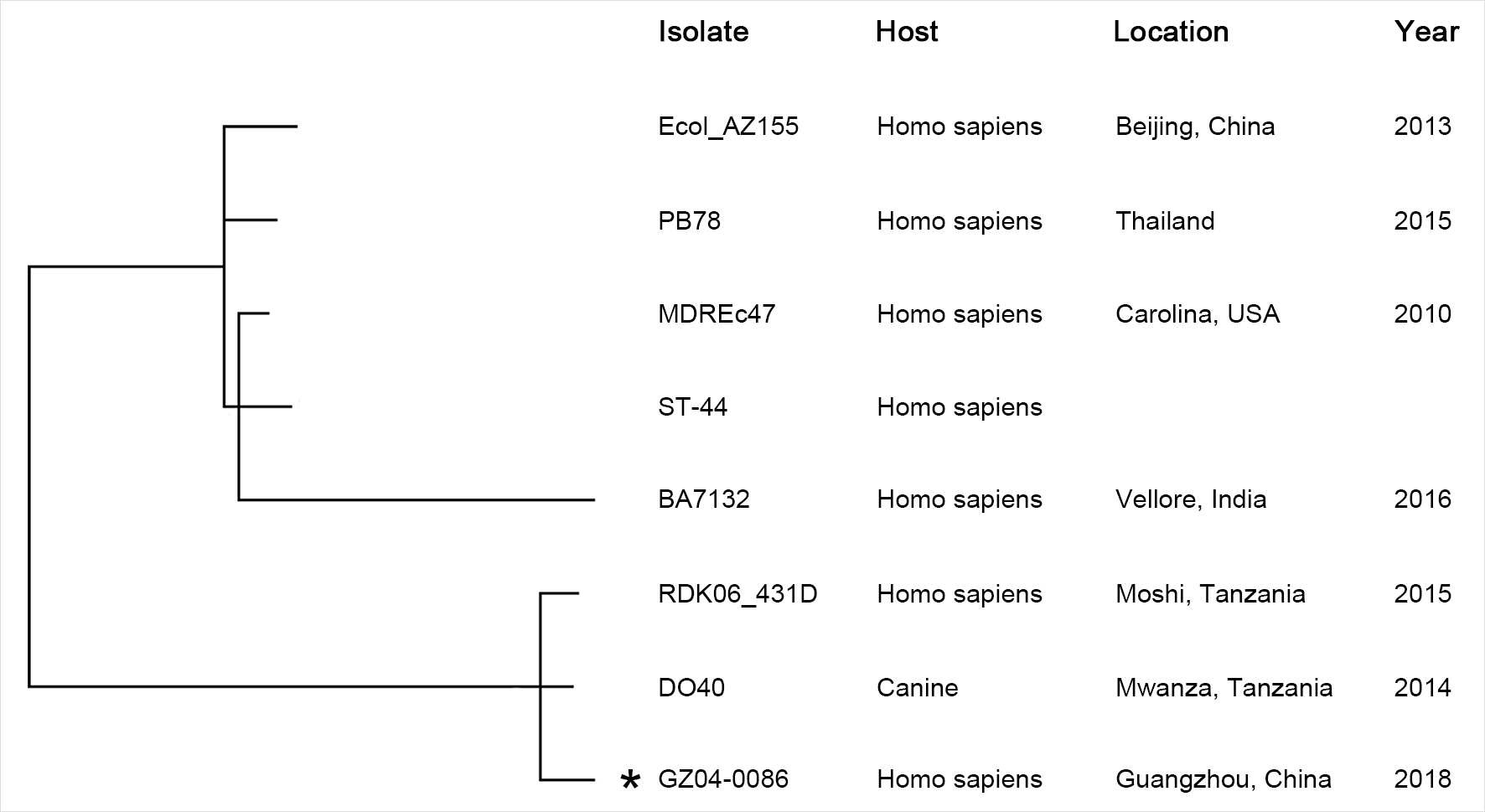
**

**Figure S2. Phylogenetic tree of *E. coli* ST44 isolates constructed by the maximum likelihood method.** High-throughput reads were simulated from genome sequences and mapped against the reference strain *E. coli* ST44 Ecol_AZ155. Whole-genome SNPs were identified and used to construct a maximum likelihood phylogenetic tree based on 1000 bootstraps. The strain GZ04-0086 in this study was marked with an asterisk.
